# Electrical Impedance Spectroscopy and Tomography for Fruit Quality Monitoring: A State-of-the-Art Analysis and Experimental Insights

**DOI:** 10.3390/s26134206

**Published:** 2026-07-03

**Authors:** Giovanni Chiorboli, Nicola Delmonte, Andrea Toscani

**Affiliations:** Department of Engineering and Architecture, University of Parma, 43124 Parma, Italy; giovanni.chiorboli@unipr.it (G.C.); nicola.delmonte@unipr.it (N.D.)

**Keywords:** bioimpedance, Cole-Cole plots, electrical impedance spectroscopy, equivalent circuit modeling, parameter estimation, food monitoring, electrical impedance tomography

## Abstract

**Highlights:**

**What are the main findings?**

- Four-electrode electrical impedance spectroscopy significantly reduces electrode polarization effects compared with conventional two-electrode configurations in fruit measurements.
- Fractional-order equivalent circuit models and impedance tomography approaches enable reliable characterization of temporal changes in fruit tissue properties during storage and ripening.

**What are the implications of the main findings?**

- Electrical impedance spectroscopy and tomography show strong potential as low-cost, non-destructive, and portable tools for real-time food quality monitoring.
- The integration of EIT/EIS techniques with automated and robotic systems may support advanced smart sensing solutions for precision agriculture and food processing applications.

**Abstract:**

Non-invasive Electrical Impedance Tomography (EIT) and Electrical Impedance Spectroscopy (EIS) are emerging as promising techniques for real-time monitoring and quality assessment in food processing and agri-food applications. This study reviews recent advances in impedance-based sensing for fruit characterization and investigates the experimental implementation of multi-electrode impedance measurements for tomographic imaging. Particular attention is devoted to electrode configurations, electrode polarization effects, and equivalent circuit modeling. Experimental measurements were performed on yellow honeydew melon samples using a four-electrode configuration and a impedance analyzer Keysight E4990 (Keysight Technologies, Santa Rosa, USA) over the frequency range from 20 Hz to 1 MHz. The impedance spectra were validated through Kramers–Kronig consistency tests and interpolated using several fractional-order equivalent circuit models, including single-Cole, double-Cole, and Hayden-based models. The results show that four-electrode measurements are less sensitive to electrode-sample interface artifacts than conventional two-electrode approaches, thereby providing a more reliable estimate of the sample impedance, particularly at low frequencies. Among the tested models, the double-Cole model provided the best interpolation accuracy, while the fractional Hayden models effectively described the temporal evolution of extracellular resistance and membrane-related parameters. Preliminary EIT reconstructions further demonstrate the feasibility of non-destructive tomographic imaging for fruit monitoring. These findings support the potential of EIS and EIT as low-cost, portable, and non-invasive tools for smart food quality assessment and precision agriculture applications.

## 1. Introduction

Non-destructive sensing techniques have become essential tools for the assessment of internal quality attributes of fresh fruit along the supply chain, from on-tree monitoring to postharvest storage and commercialization [1,2,3]. Advanced methods such as Nuclear Magnetic Resonance (NMR) [4] and Magnetic Resonance Imaging (MRI) [5] enable detailed characterization of internal structure, water distribution, and tissue heterogeneity, providing valuable information on physiological disorders and textural properties. Optical techniques, including chlorophyll fluorescence and Delayed Light Emission (DLE) [6], are widely applied to probe photosynthetic activity and detect early stress conditions or senescence-related changes. In parallel, spectroscopic approaches—particularly Near-InfraRed Spectroscopy (NIRS) [7]—have been extensively employed to estimate chemical constituents such as soluble solids, water content, and acidity through the interaction of electromagnetic radiation with molecular bonds. Furthermore, hyperspectral imaging and machine vision systems integrate spatial and spectral information, allowing simultaneous evaluation of external and internal quality parameters [8]. Despite their proven effectiveness, these techniques often require sophisticated instrumentation, complex data processing, and may be limited by penetration depth or sensitivity to surface properties.

Within this framework, Electrochemical Impedance Spectroscopy (EIS) has emerged as a promising complementary technique for non-destructive quality assessment of biological tissues [9,10]. EIS typically involves applying a small alternating electrical excitation to the sample, gradually varying the frequency of the sinusoidal signal (for instance, a voltage), and measuring the corresponding sinusoidal current, so that the electrochemical impedance can be calculated for each frequency across a wide frequency range. In fact, fruit contains free and bound charges that, in response to an applied electric field, move and rearrange themselves, generating conduction and polarization currents. Unlike conduction currents, polarization currents are continuously reversed by the alternating excitation signal and therefore do not result in a permanent charge displacement. As a consequence, the impedance frequency response reflects the dielectric and conductive properties of the sample [11,12].

**Figure 1:**
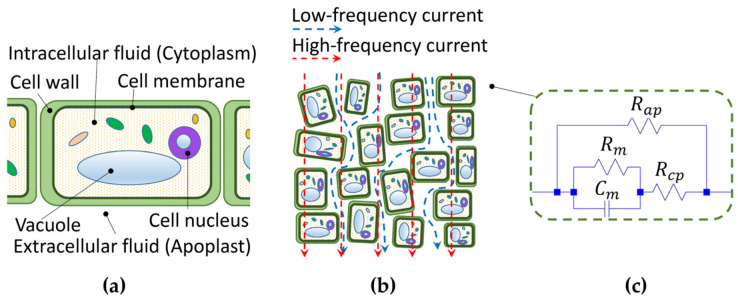
Biophysical model of plant tissue and its equivalent electrical circuit used in EIS. (**a**) Simplified structure of a plant cell. (**b**) Low- and high-frequency current paths. (**c**) A possible equivalent electrical circuit (Hayden model).

**Figure 2:**
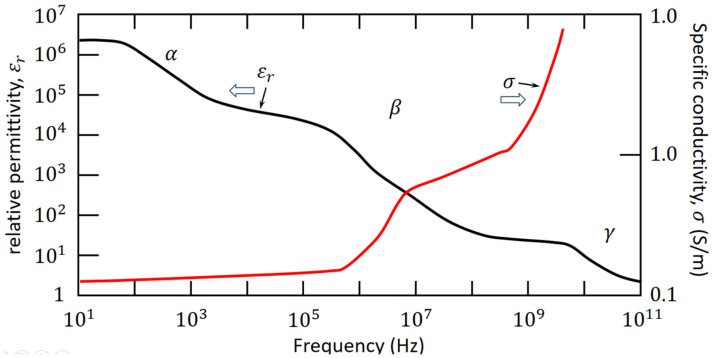
α, β, and γ dispersions of the dielectric constant *ε_r_* and conductivity *σ* as function of frequency. The open arrows indicate which y-axis each quantity refers to.

From a biophysical perspective, plant tissues can be represented by equivalent electrical circuits, where intracellular and extracellular fluids contribute to resistive components (*R_cp_*, *R_ap_*), while cell membranes exhibit capacitive behavior (*C_m_* in parallel with *R_m_*) due to charge accumulation across lipid bilayers (see Figure 1). Consequently, impedance spectrum is strongly influenced by cell structure, membrane integrity, ionic content, and water distribution. Furthermore, the current distribution within plant tissues is strongly frequency-dependent, with extracellular pathways dominating at low frequencies and transmembrane conduction becoming significant at higher frequencies. The impedance response, which occurs in a series of steps, can be interpreted in terms of dispersion phenomena typically classified as α-, β- and γ-dispersions, as shown in Figure 2 and described in detail in [13]. Each step reflects the loss of a particular polarization mechanism and the associated change in current pathways as the frequency increases. The α-dispersion, occurring at low frequencies (Hz–kHz), is mainly associated with ionic diffusion and charge redistribution phenomena in the extracellular space, where the current is confined to the apoplastic pathway due to the high capacitive reactance of cell membranes [14]. At intermediate frequencies (kHz–MHz), the β-dispersion dominates and reflects the dielectric properties of cell membranes, whose capacitive behavior progressively decreases with increasing frequency, allowing current penetration into the intracellular compartment [15]. At higher frequencies (MHz–GHz), the γ and δ-dispersions are related to dipolar relaxation processes, primarily associated with water molecules (δ-dispersion) and bound charges within the tissue matrix [10].

In plant tissues, these mechanisms result in a complex impedance spectrum that reflects the combined contribution of extracellular fluids, intracellular fluids, and membrane interfaces, which can be effectively modeled using distributed resistor–capacitor networks [10]. The measured impedance therefore depends on several factors, including tissue structure, cell integrity, ionic concentration, and water distribution, all of which evolve during ripening, senescence, or stress conditions. In particular, membrane permeability and structural degradation significantly affect the capacitive component, while changes in electrolyte content and compartmentalization influence the resistive pathways [14].

**Table 1:** Comparison of recent non-disruptive EIS papers in the range of β-dispersion.

Ref.	Fruit	Objective	Instrument	Frequency Range	Signal	Number of elect.	Electrode
[16]	banana, lemon, cucumber, orange, mandarin	n.a.	Biologic SP150	200 mHz–200 kHz	353 mV	2	Ag/AgCl
[17]	banana	ripening	Keysight 4294A	50 Hz–1 MHz	1 mA	2	Ag/AgCl
[18]	mandarin orange	ripening	Keysight 4294A	50 Hz–100 MHz	1 mA	2	Ag/AgCl
[19]	mango	ripening	Metrohm AUT302N	1 Hz–1 MHz	100 mV	2	Ag/AgCl
[20]	strawberry	ripening	Solartron 1260	1 Hz–1 MHz	1 Vrms	4	n.a.
[21]	avocado	ripening	AD5933	5 kHz–15 kHz	1 Vpp	2	Ag/AgCl
[22]	lemon	freeze-damage	Prototype	1 Hz–1 MHz	<500 mV	2	double-needle
[23]	apple, banana, cucumber	n.a.	Prototype	100 Hz–100 kHz	115 µA	4	Ag/AgCl
[24]	apple, banana	ripening	AD5933	100 Hz–85 kHz	1 Vpp	2	Ag/AgCl
[25]	grapefruit	freeze-damage	Prototype	100 Hz–1 MHz	1 Vpp	2	double-needle
[26]	blueberry, plum	ripening	IM3570	4 Hz–4 MHz	1 Vpp	2	needle
[27]	avocado	ripening	AD5933	4 Hz–4 MHz	0.5 V	2	Ag/AgCl
[28]	citrus fruit	mechanical damage	IM3570	4 Hz–4 MHz	0.5 V	2	Ag/AgCl
[29]	minikiwi	mechanical damage	IM3570	4 Hz–1 MHz	1 V	2	Flexible electrodes
[30]	yellow pitaya	ripening	TH2829LX	1 kHz–100 kHz	n.a.	2	Ag/AgCl
[31]	apple, banana, pumpkin	freshness	Analog Discovery 3	50 Hz–1 MHz	n.a.	2	Ag/AgCl
[32]	pear	ripening	IM3570	10 Hz–1 MHz	0.5 V	2	Conductive hydrogel/AgCl
[33]	banana	ripening	DSO + AWG	1 kHz–10 MHz	n.a.	2	n.a.
[34]	apple	internal browning	Analog Discovery 2	10 Hz–10 MHz	n.a.	2	Ag/AgCl
[35]	guava	ripening	AD5933	1 kHz–100 kHz	n.a.	2	Ag/AgCl or needle

Consequently, EIS provides a frequency-resolved electrical fingerprint of fruit tissues, enabling the discrimination of physiological states such as ripening stage, mechanical damage, or chilling injury through the analysis of impedance magnitude and phase over a broad frequency range.

In many recent studies, these impedance spectra are further processed using machine learning and artificial intelligence techniques for classification, regression, and quality prediction tasks. Although these approaches have shown considerable promise, a detailed review of machine learning methodologies is beyond the scope of the present work, which focuses primarily on measurement principles, instrumentation, and impedance modeling aspects.

Compared to conventional analytical techniques, EIS offers several advantages, including rapid measurement, low cost, portability, and minimal sample preparation, making it particularly suitable for in situ and real-time monitoring applications in the agri-food sector. EIS can be destructive when applied to a slice of fruit [36,37], or non-destructive, but also contact-based, using electrodes, or contactless, using methods such as capacitive impedance analysis [38] or magnetic induction spectroscopy [39]. Limiting to non-destructive and contact techniques, which can be employed in the field with the aid of robotic arms [40,41], Table 1 lists some of the articles that, over the past 10 years, have applied EIS to fruit analysis in the frequency range corresponding to β-dispersion.

The used instruments range from automatic balance bridges and LCR meters—which are the most accurate—to potentiostats/galvanostats [16,19] and frequency response analyzers [20], all of which are reference-grade laboratory instruments, as well as prototypes that still rely on more commonly used laboratory instruments and AFEs (Analog Front-Ends), up to truly portable multifunctional [35] or dedicated [23] measurement devices or an integrated circuit, such as AD5933, which can be viewed as a simplified version of a frequency response analyzer for impedance measurements. In most cases, two—and sometimes four—disposable Ag/AgCl adhesive ECG electrodes are used, each equipped with an integrated drop of electro-gel that ensures a stable and reliable lead. These are best suited for in situ monitoring. For more field-based activities, where integrated detection and harvesting with flexible robotic manipulators are required, needle-type electrodes can be used; however, since they penetrate the sample, they damage the fruit during measurement. Alternatively, specially developed flexible, reusable wearable electrodes can be used [29,42].

Closely related to EIS is Electrical Impedance Tomography (EIT). Although EIS and EIT pursue different objectives, they share the same physical basis, namely the measurement of electrical impedance in biological tissues through externally applied currents and measured voltages. EIS primarily aims to characterize the frequency-dependent impedance response of a sample, whereas EIT seeks to reconstruct the spatial distribution of electrical properties within the sample. In this sense, EIT can be regarded as a spatial extension of impedance measurements and can be implemented using instrumentation and electrode configurations closely related to those employed in EIS. While EIT is often performed at a single frequency, multifrequency EIT systems have also been proposed, further strengthening the connection between the two techniques.

Its goal is to determine the distribution of electrical impedance within a given domain by injecting currents and measuring voltages (or vice versa) using a series of electrodes positioned non-invasively in contact with the boundary of the domain, such as the circumference of a fruit [43,44,45,46]. EIT can use the same instrumentation as EIS combined with a matrix of switches that allows the selection, within an array of different electrodes placed around the circumference of the fruit, of those used for current injection and those for voltage measurement. The EIT technique has the advantage of being non-invasive and offering good temporal resolution, which could make it suitable for studying ripening or aging on the tree or on the shelf, or for assessing mechanical or cold damage. However, in addition to being known for its poor spatial resolution, for these types of applications it is necessary to address the issues related to the electrodes’ sensitivity to movement and the quality of contact.

The relatively limited number of EIT studies dedicated to fruit quality assessment can be partly explained by several technical challenges. Unlike medical applications, fruits generally exhibit relatively small conductivity contrasts during physiological processes such as ripening, while the changes are often distributed throughout the tissue rather than confined to well-localized regions. In addition, EIT suffers from inherently low spatial resolution and requires the solution of an ill-posed inverse problem, making image reconstruction sensitive to measurement noise, electrode positioning, and contact conditions. As a result, conventional impedance spectroscopy often provides a simpler and more robust approach for monitoring global fruit quality changes, whereas EIT appears particularly suited for the detection and localization of internal defects or localized tissue alterations.

Some studies [47,48] consider a tank filled with water and pieces of vegetables, such as carrots, potatoes, and cucumbers. These studies are not yet applicable to field measurements. Kumar et al. [44] perform some EIT measurements on a papaya, primarily to compare certain image reconstruction algorithms, but without reaching conclusions applicable to the analysis of ripening or mechanical or cold damage. San-Pablo-Juarez et al. [45] state in the title and abstract that they can determine fruit ripeness from EIT images, but do not propose a series of measurements over time to assess its evolution. Finally, Almanzor et al. [46] apply impedance tomography electrodes to a soft robotic gripper, but do not reconstruct an image; rather, they use the electrodes to obtain numerous pseudo-impedance measurements from different directions to serve as features for a machine learning algorithm.

Non-invasive analyses of fresh fruit are also characteristic of the field of microwave spectroscopy, both as contact microwave spectroscopy [49] and as a contactless technique that enables the reconstruction of microwave images using tomographic techniques [50]. Bilgin et al. [51] propose a recent review of the state of the art of microwave spectroscopy and imaging.

The paper is organized as follows. Section 2 presents the materials and methods adopted for both EIS and EIT, including the measurement configurations, equivalent circuit models, experimental setup, and image reconstruction procedures. Section 3 reports the experimental results obtained on yellow honeydew melon samples, discussing the impedance evolution during storage, the performance of different fractional-order models, and preliminary tomographic reconstructions. Section 4 discusses the implications of the results in relation to previous studies and highlights the main advantages and limitations of impedance-based techniques for fruit quality monitoring. Finally, Section 5 summarizes the main conclusions and outlines possible future developments for portable and smart sensing applications.

## 2. Materials and Methods

### 2.1. Electrical Impedance Spectroscopy (EIS)

The non-destructive measurements listed in Table 1 always refer to 2-electrode or 4-electrode measurements, regardless of the instrument used. For bioimpedance measurements, the tetrapolar (4-electrode) configuration has long been recognized as the preferred approach because it separates current injection from voltage sensing, thereby minimizing the influence of electrode polarization, intended here as the electrode–sample interface impedance in the classical sense of Schwan [52]. This measurement principle was introduced several decades ago [53,54] and remains the reference method for accurate impedance characterization of biological tissues. Nevertheless, two-electrode configurations are still widely employed in portable and low-cost systems, although their measurements may be significantly affected by interface artifacts, particularly at low frequencies.

**Figure 3:**
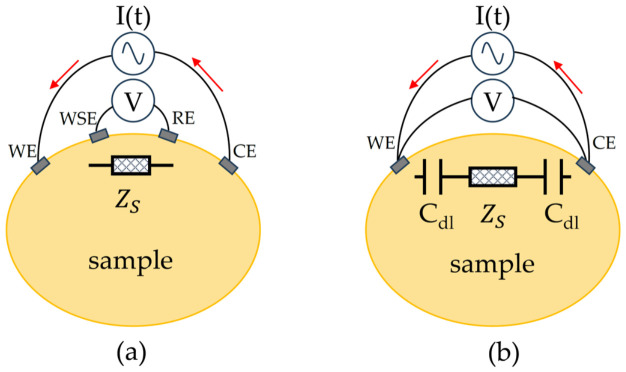
Typical measurement setup. (**a**) Four-electrode setup and (**b**) two-electrode setup.

Four-electrode EIS measurements are typically performed in galvanostatic mode, with a current stimulus applied between the outer electrodes—the Working Electrode (WE or HI) and the Counter Electrode (CE or LI)—and the voltage measured between the inner electrodes—the Working Sensing Electrode (WSE or HV) and the Reference Electrode (RE or LV), as shown in Figure 3. In the two-electrode configuration, the high-current and high-potential terminals are connected together on one side, and the low-current and low-potential terminals on the other. In this case, one often operates in potentiostatic mode as well, that is, by applying a sinusoidal voltage and measuring the current.

Various materials are used for electrodes, but the tests listed in Table 1 most often refer to Ag/AgCl electrodes (a layer of silver coated with silver chloride) because they are readily available and, above all, because they are sufficiently non-polarizable in DC [55].

In fact, a double layer forms at the electrode-electrolyte interface due to ion exchange between the metal and the electrolyte; this can be viewed as a series combination of the half-cell resistance and the double-layer capacitance, with both the resistance and capacitance being frequency-dependent. In two-terminal measurements, the measured impedance includes both the sample response and the contribution of the electrode interfaces, including contact impedance, double-layer capacitance and possible charge-transfer processes at the Ag/AgCl electrodes. These contributions are particularly relevant at low frequencies and may lead to a poorly conditioned estimate of the sample impedance. In the four-terminal configuration, the voltage-sensing electrodes draw negligible current and therefore develop negligible polarization voltage, so the measured voltage drop is more representative of the fruit tissue. Since no clear phase-angle feature attributable to finite-length diffusion was observed above 20 Hz, the main role of the four-terminal configuration was to minimize electrode-sample interface effects rather than to correct for a specific diffusion-controlled process [52,56,57]. Polarization depends on current density, which should remain below 0.1 mA/cm^2^ to ensure a linear regime; this limit increases to 1 mA/cm^2^ at frequencies between 100 Hz and 1 kHz [52]. If typical ECG electrodes with a diameter of 8–10 mm are used, currents on the order of 50 or 100 μA should be employed, at least at low frequencies, to avoid distortion and thus harmonics. Depending on the type of fruit, the impedance can be expected to range between 100 Ω and 10 kΩ, which allows the voltage limit to be defined when driving in potentiometric mode.

**Figure 4:**
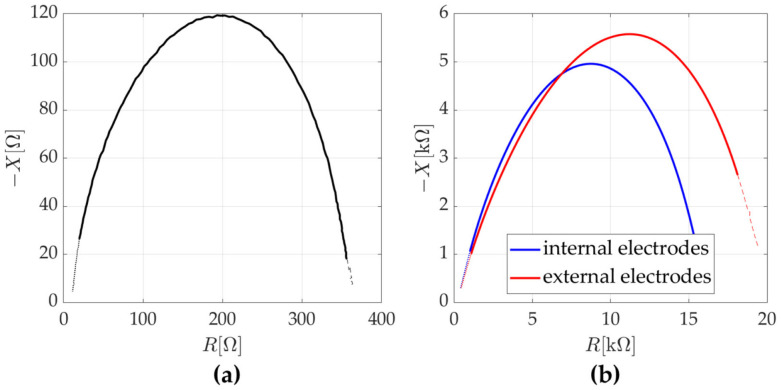
Cole-Cole plot of a yellow honeydew melon from 100 Hz to 100 kHz (solid lines). Dashed lines and dotted lines correspond to the frequency ranges from 20 Hz to 100 Hz and from 100 kHz to 1 MHz, respectively. (**a**) Four-electrode setup. (**b**) Two-electrode setup.

The well-known electrode polarization is often overlooked, and it is not considered that the impedance of the electrode-sample interface can be much higher than the sample impedance and that, moreover, it can also change over time. As an example, Figure 4 shows the Cole-Cole plot obtained using the two setups during the measurement of a yellow honeydew melon by the impedance analyzer Keysight E4990 (Keysight Technologies, Santa Rosa, CA, USA) from 20 Hz to 1 MHz. The figure is intended to compare the impedance obtained with the four-electrode configuration (black curve) and the impedance obtained with two-electrode measurements (red and blue curves) performed using electrode pairs belonging to the same electrode arrangement. This comparison highlights the substantial differences between the two measurement configurations, particularly at low frequencies, where electrode–sample interface effects and other measurement-system contributions become more significant. In the four-terminal configuration, the maximum value of the reactive component was about 120 Ω, whereas values of approximately 5.6 kΩ and 5 kΩ were obtained using the external and internal electrode pairs in two-terminal mode, respectively. The much larger reactive response observed in the two-terminal measurements indicates that the measured impedance is strongly affected by electrode–sample interface and contact contributions, rather than by the intrinsic impedance of the fruit tissue alone. Consequently, the sensitivity of the two-terminal configuration to ageing-related changes in the sample bulk impedance is reduced, since relatively small variations in the tissue response may be masked by the larger and less reproducible interfacial contribution. For this reason, the four-terminal configuration was adopted for the subsequent analysis.

An array of four Ag/AgCl electrodes was attached in a line around the circumference of the melon, spaced 45° apart (~ 6 cm). In the case of the two-electrode measurement, the impedance between the first and fourth electrodes, as well as that between the second and third electrodes, was measured using a potentiometric technique, applying a voltage of 500 mV, in the constant voltage mode. The impedance at 20 Hz was 19 kΩ and 16 kΩ, when measured on the external electrodes and internal electrodes, respectively. In the four-electrode measurement, the impedance at 20 Hz drops to 350 Ω, which is more than one order of magnitude lower than that obtained in the two-electrode measurement mode. The values obtained for melon tend to be low due to the fruit’s high water content [23], but even for fruits with lower water content, the difference between the two-electrode and four-electrode measurements can exceed 100%, raising some doubts about the results reported in various studies, especially considering that the impedance between the electrodes and the fruit can also vary over time.

In addition, in several studies, measurements were performed using the AD5933 with a DC bias of 0.76 V, which results in an even more significant polarization contribution from the interface.

Finally, to verify the validity of the experimental data, one can apply the Kramer-Kronig relations, which allow the real part of the complex impedance to be calculated from the imaginary part, and the imaginary part to be calculated from the real part alone. For this to be possible, four criteria—linearity, causality, stability, and finiteness—must be met [12].

Once the complex impedance has been measured over a wide frequency range using a two- or four-electrode setup, the experimental data are commonly interpolated with the data that would be obtained from an equivalent circuit model. Biological tissues behave, as a first approximation, as electrolytes because of the ions they contain. This can be described using the Debye model, in which the infinite resistance $R_{\infty }$ is connected in series with a parallel combination of a capacitance C and a resistance $R_{1}=\left(R_{0}-R_{\infty }\right)$, resulting in the complex impedance

(1) $$Z\left(\omega \right)=R_{\infty }+\frac{R_{0}-R_{\infty }}{1+j\omega \tau }=R\left(\omega \right)+jX\left(\omega \right)$$

where $\tau =CR_{1}$. If an impedance of this type is driven by a current step, the measured voltage exhibits a classic exponential decay with a time constant of τ. If we plot this impedance in the complex plane as -X versus R (the Cole-Cole plot), we can see a semicircle that moves counterclockwise as the frequency increases from $R_{0}$ to $R_{\infty }$. However, it was soon observed that, in the time domain, the relaxation is not exponential, and the Cole-Cole plot shows a downward-sloping curve; consequently, the Cole model was introduced, which describes the relaxation using a fractional power law

(2) $$Z\left(\omega \right)=R_{\infty }+\frac{R_{0}-R_{\infty }}{1+{\left(j\omega \tau \right)}^{\alpha }}$$

where α (0≤α≤1) is related to the distribution of relaxation times. In this model, the constant-phase element (CPE) replaces the capacitance, and its admittance is $Y_{CPE}={\left(j\omega C\right)}^{\alpha }$.

Since biological tissues exhibit behaviors that can be described using classical circuit components with lumped parameters, such as resistors (e.g., extracellular and intracellular fluids) and capacitors (the cell membrane), or as distributed components that can be viewed as composed of an infinite number of lumped-parameter components, such as CPEs, which are better suited to describing a fractal structure and morphology, different circuit models can be used to interpolate the experimental data [55]. Just as when selecting the signal level to apply to the electrodes, a choice must be made that strikes an acceptable balance between ensuring a reasonable signal-to-noise ratio and guaranteeing that the system remains in the linear region; so too, in the case of the equivalent circuit, one must find a circuit model that allows for reasonably good interpolation of the experimental data, but that is also not too complex so as to meaningfully represent certain elements of the sample’s physical structure. For example, CPE often replaces the capacitance used to model the membrane because, on the one hand, a single capacitance is not sufficient to adequately represent the behavior of the cell membrane, and on the other hand, the mathematical model corresponding to a double- or triple-layered membrane is more complex than that provided by a single CPE component [15]. Warburg-type elements are commonly used in two-electrode measurements to describe diffusion-controlled processes at the electrode–sample interface [24,58]. We agree that such processes are not physically eliminated by the four-electrode configuration. However, their contribution to the measured voltage is strongly reduced, because the voltage-sensing electrodes draw negligible current, whereas most of the electrode polarization and diffusion-related voltage drop occurs at the current-injecting electrodes. Since no clear phase-angle feature attributable to finite-length diffusion was observed in the investigated frequency range, an explicit Warburg element was not included. Instead, non-ideal dispersive effects were described phenomenologically by CPEs, which also include Warburg-like behavior as a limiting case when the exponent approaches 0.5. The circuits we will be discussing are all fractional-mode circuits, because they allow for better data interpolation [16,23].

We will refer to the single-dispersion and double-dispersion circuits (Cole impedance model) and to the fractional Hayden and simplified Hayden models [16,24].

**Figure 5:**
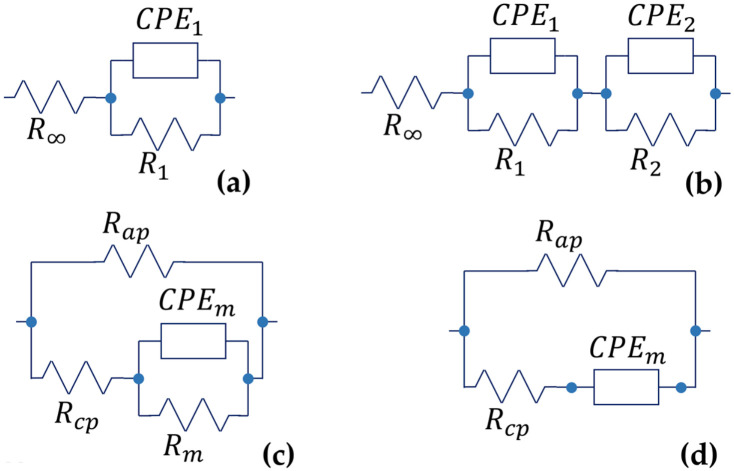
Equivalent circuit models: (**a**) single-dispersion or single-Cole; (**b**) double-dispersion or double-Cole; (**c**) fractional Hayden; (**d**) fractional simplified Hayden or Fricke-Morse.

The equivalent circuit models considered in this work are shown in Figure 5. The single-Cole and double-Cole models are two widely used methods for characterizing the impedance of biological tissues. The former uses only four parameters to describe the sample’s impedance, while the latter requires seven. Hayden’s model, originally an integer model, provides a more accurate description of the various components of biological tissue. The current flows in parallel through the extracellular fluid (the apoplast, $R_{ap}$) and through the cell wall (a capacitance $C_{m}$ in parallel with a resistance $R_{m}$) and intracellular (or cytoplasm) fluid ($R_{cp}$). In the fractional model, the CPE replaces $C_{m}$ and the model requires five parameters. In the fractional simplified Hayden model, it is assumed that the resistance $R_{m}$ is very large, so as not to produce any effects in the frequency range under consideration, and the number of parameters is reduced to four. Another model that is often used is the Zhang model, or the double-shell model, in either its integer or fractional form [22], with five and seven parameters, respectively. In the case of the melon, this model yields significantly worse results than the proposed ones, and will not be considered further.

The measurements were performed using the impedence analyzer Keysight E4990 (Keysight Technologies, Santa Rosa, CA, USA) in galvanostatic mode with I = 200 μA. A total of 201 impedance values were recorded between 20 Hz and 1 MHz on a logarithmic scale, and the experimental data were interpolated between 100 Hz and 100 kHz. For the interpolation, Powell’s dog-leg method was used to minimize the error; for each measurement dataset, all model parameters were estimated simultaneously through nonlinear least-squares optimization of the complex impedance spectrum. The fitting procedure minimized the difference between the measured and calculated real and imaginary impedance components according to:

(3) $$r_{1}^{2}=\sum_{1=1}^{N}\left[{\left(R_{i,m}-R_{i,c}\right)}^{2}+{\left(X_{i,m}-X_{i,c}\right)}^{2}\right]$$

where $R_{i,m}$ represents the real part of the impedance measured at the *i*-*th* frequency, $R_{i,c}$ represents the real part of the impedance of the equivalent circuit, and $X_{i,m}$ and $X_{i,c}$ represent the corresponding imaginary parts. To evaluate the quality of the models, we used the root mean square error

(4) $${RMSE}_{1}=\sqrt{r_{1}^{2}/\left(N-p\right)}$$

where *N* and *p* denote, respectively, the number of data points and the number of model parameters. Since the impedance varies by more than an order of magnitude as the frequency changes, and the absolute value loses some of its meaning, the percentage root mean square errors are also reported:

(5) $${RMSE}_{2}=\sqrt{\frac{1}{N-p}\sum_{1=1}^{N}\left[\frac{{\left(R_{i,m}-R_{i,c}\right)}^{2}+{\left(X_{i,m}-X_{i,c}\right)}^{2}}{R_{i,m}^{2}+X_{i,m}^{2}}\right]}\;{RMSE}_{3}=\sqrt{\frac{1}{N-p}\sum_{1=1}^{N}\left[\frac{{\left(R_{i,m}-R_{i,c}\right)}^{2}}{R_{i,m}^{2}}+\frac{{\left(X_{i,m}-X_{i,c}\right)}^{2}}{X_{i,m}^{2}}\right]}$$

### 2.2. Electrical Impedance Tomography (EIT)

**Figure 6:**
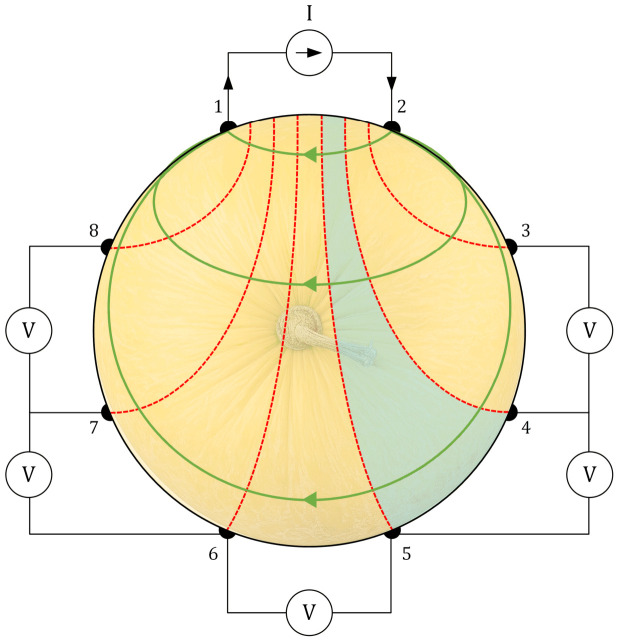
Electrode arrangement, electric field line patterns, and current distribution for eight electrodes sensing.

The main goal of an EIT inverse problem solver is to obtain the internal fruit conductivity distribution analyzing a series of measured voltages. These voltages can be easily obtained from a series of impedance measurements. Therefore, the hardware used in the EIT always includes the Keysight E4990A impedance analyzer, which always functions as a sinusoidal current source, now at frequencies between 1 kHz and 100 kHz. $N_{e}=8$ Ag/AgCl ECG electrodes were used, evenly spaced around the circumference of the melon, in a configuration like that shown in Figure 6. This was considered a good compromise between spatial resolution, acquisition time, and the complexity of potential portable hardware for field measurements [46].

**Figure 7:**
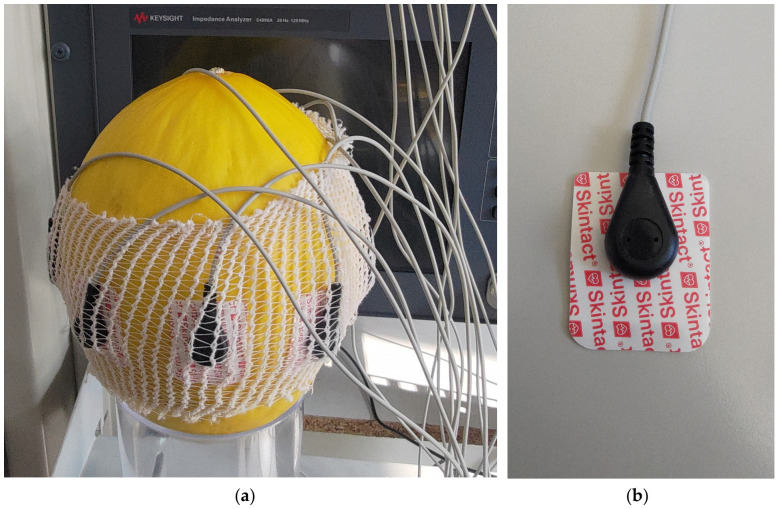
Measurement setup. The yellow honeydew melon the eight electrodes, and the mesh used to keep the electrodes and wiring in place (**a**); the Ag/AgCl electrode together with its connector (**b**).

We used a RIGOL M302 modular data acquisition/switch system together with a MC3648 matrix module to select the electrodes for current injection and those for voltage sensing. The protocol chosen was injection and measurement between adjacent electrodes: as shown in Figure 6 and Figure 7, current is initially injected between electrodes 2 and 1, and the voltage is measured between the remaining adjacent pairs (from 3 and 4 to 7 and 8); then, the current injection electrodes are moved to positions 3 and 2 and the sensing electrodes from 4 and 5 to 8 and 1, until a vector of $N_{e}\times \left(N_{e}-3\right)=40$ complex pseudo-impedance values is obtained, since the electrodes used for current injection are not used for voltage sensing. This strategy of stimulating with one pair of adjacent electrodes and measuring with another adjacent pair offers greater sensitivity near the electrodes, whereas the strategy of stimulating with opposite pairs and measuring with adjacent pairs is generally considered to provide more uniform sensitivity across the domain [59]; however, in this case, the pseudo-impedance vector is reduced to $N_{e}\times \left(N_{e}-4\right)=32$ elements. In both cases, similarly to four-electrode EIS measurements, the influence of electrode-interface impedance and other measurement-system contributions on the estimated sample impedance is reduced because the voltage-sensing electrodes carry negligible current.

The inverse reconstruction problem is typically ill-posed and subject to measurement noise, incomplete electrode distribution, and modeling errors; for this reason, differential EIT is generally preferred, as it is considered more robust than absolute EIT [60]. In differential EIT, two datasets are compared, and only the change in impedance is reconstructed. This makes the reconstruction more robust to systematic errors, since many uncertainties cancel each other out in the difference.

The images were then reconstructed using EIDORS (version 3.12), an open-source software widely used to reconstruct images from electrical impedance tomography data. The one-step Gauss-Newton differential inversion algorithm was employed [61], as well as the NOSER prior as a regularization term to make the solution stable and less sensitive to noise [62].

## 3. Experimental Results

### 3.1. Electrical Impedance Spectroscopy (EIS)

To verify that the impedance spectrum consistently corresponded to a linear time-invariant system, the Lin-KK v. 1.3 software was used to apply the Kramer-König transformations, consistently yielding residuals of less than 1% in the 100 Hz–1 MHz range.

**Figure 8:**
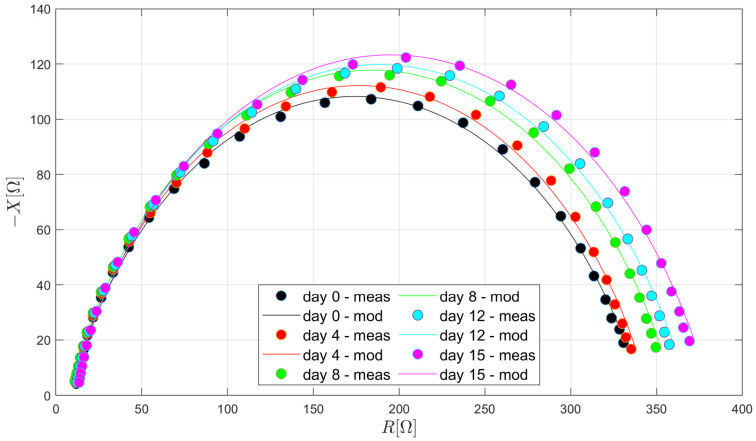
Cole-Cole plot of the yellow melon: The markers correspond to the experimental data, and the solid lines correspond to the data obtained by interpolation using the simplified fractional Hayden model.

The impedance of a yellow honeydew melon (Cucumis melo inodorus) was measured over a period of 15 days at a room temperature of 24 °C ± 1 °C. Figure 8 shows the Cole-Cole plots obtained from day 0 to day 15, which confirms a change over time observable in this frequency range.

The experiments were repeated on five different melons, yielding similar qualitative trends. For clarity, the results reported in the following correspond to a representative specimen. Owing to the limited number of samples, the present study should be regarded as a methodological and proof-of-concept investigation rather than a statistically comprehensive analysis of fruit-to-fruit variability.

The experimental data acquired on different days during the 15-day storage period were independently fitted using the four equivalent circuit models. For each measurement day, the model parameters and the corresponding RMSE values were estimated separately. Table 2 summarizes the mean RMSE values obtained over all measurement days, while the values reported in parentheses represent the corresponding standard deviations. As can be seen, the seven-parameter double-Cole model is the one that, on average across different days, best fits the data.

Although the double-Cole model provided the best numerical agreement with the experimental Cole-Cole plots, its larger number of fitting parameters did not lead to a clearer physical or phenomenological interpretation of the ageing process. None of the seven parameters extracted from the double-Cole model showed a statistically meaningful evolution with storage time, as the corresponding regression coefficients were always below $r^{2}=0.9$. This suggests that the improved fitting accuracy of the double-Cole model is mainly associated with its higher flexibility, rather than with a more robust description of the time-dependent changes occurring in the fruit tissue. Therefore, although the double-Cole model remains useful for accurately reproducing the shape of individual Cole-Cole plots, it appears less suitable for extracting ageing markers from the present dataset.

Conversely, the single-Cole, fractional Hayden, and simplified fractional Hayden models, despite using fewer fitting parameters, provided a more consistent description of the temporal evolution of the electrical properties. For this reason, the subsequent analysis focuses mainly on these models that offer a better compromise between fitting accuracy, number of free parameters, and interpretability of the ageing-related trends. In particular, the systematic increase in some resistive parameters and the exponential decrease in the CPE parameters provide a compact and reproducible description of the changes in the impedance response of fresh fruit during storage.

**Figure 9:**
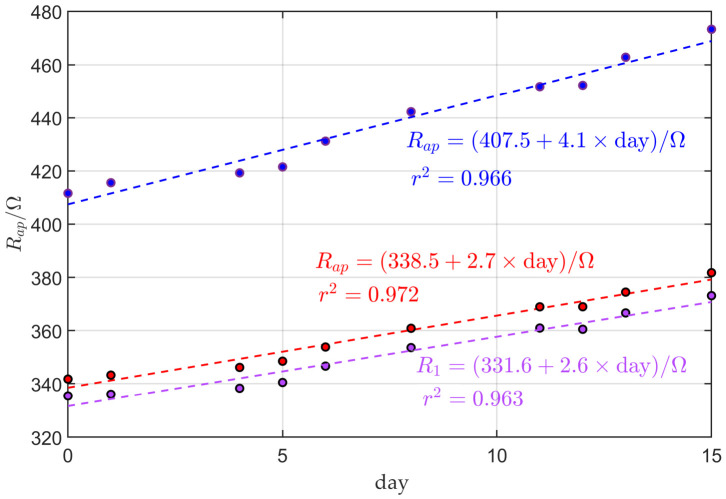
Evolution of the extracellular fluid resistance in the simplified fractional Hayden model (red) or in the fractional Hayden model (blue) and of R_1_ in the single-Cole model (violet).

**Figure 10:**
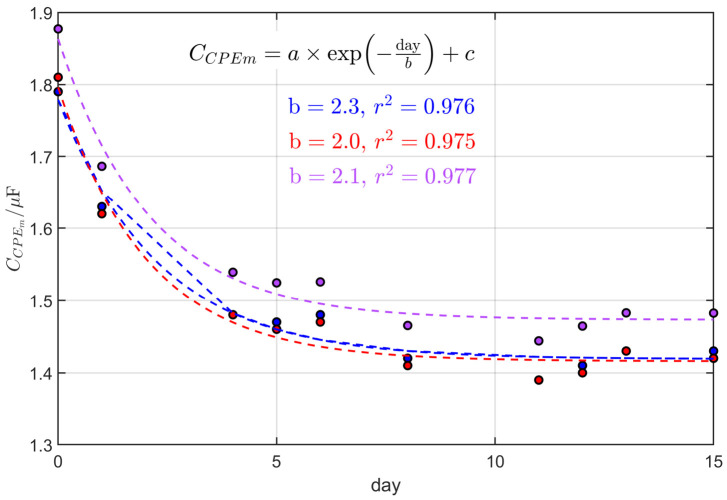
The trend in CPE indicates a change in the cell membrane as well, whose capacity decreases with a time constant of approximately 2 days, for Hayden’s model (blue), the simplified version (red), and for the single-Cole model (violet).

The resistance of the extracellular fluid $R_{ap}$ shows a linear trend over the 15 days, according to both the Hayden model and the simplified version, and similar for $R_{1}$ in the single-Cole model, as shown in Figure 9. The ${CPE}_{m}$ value also shows a trend, this time an exponential decay with a time constant of approximately 2 days, in all considered models (Figure 10). The dispersion constant of ${CPE}_{m}$ remains approximately constant at 0.75, as do the resistances of the intracellular fluid, $R_{cp}≅8\;\Omega$, and that of the membrane, $R_{m}≅2\;k\Omega$, in the Hayden model. Similarly to $R_{cp}$, the $R_{\infty }$ series resistance in the single-Cole model also remained small of the order of 7 Ω and did not exhibit a significant trend with time.

### 3.2. Electrical Impedance Tomography (EIT)

As noted in the Introduction, to the authors’ knowledge, there are not many studies on the reconstruction of images inside fruit that include any form of validation: perhaps the most interesting is the study by Verma et al. [63], in which inhomogeneities were created in half a watermelon by inserting a piece of cucumber—which is more conductive—or a piece of plastic into the pulp. If the goal were simply to study fruit ripening, we have seen that it would be advisable to simply use EIS, possibly repeating the measurement across multiple electrodes.

The EIT technique appears to be more effective for identifying and locating damage confined to a specific region. For this reason, we decided to acquire tomographic images after injecting 1 cc of saline solution immediately above electrode 1, inserting the needle to a depth of 1 cm.

**Figure 11:**
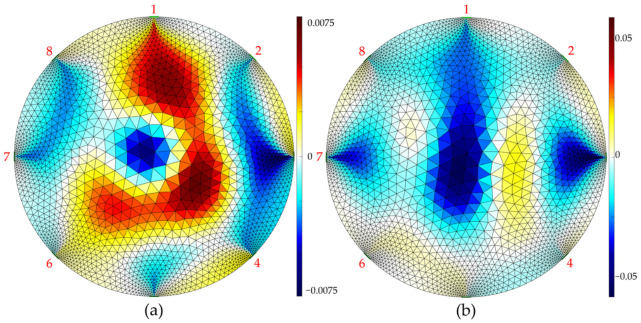
Conductivity difference shortly after injecting 1 cm^3^ of saltwater under electrode 1 (**a**); conductivity difference after 4 days (**b**). Images reconstructed from measurements acquired at 69.8 kHz.

As mentioned in Section 3.2, the measurement was made by calculating the difference from the pseudo-impedance data measured prior to the injection. Figure 11a shows the resulting image half an hour after injection, and Figure 11b shows the image four days later. Half an hour after injection, the effect is not yet clear; it appears that something has occurred near electrode 1, but the change in conductivity is very small and seems to indicate a slight reduction. After four days, the image reveals an increase deep beneath electrode 1. Note the difference in color scale between the two figures. The saltwater has spread beneath the electrode, altering the conductivity of the pulp—a phenomenon that can be observed by cutting the melon open. The images shown in Figure 11 were reconstructed from measurements acquired at 69.8 kHz. Preliminary observations indicated that the conductivity perturbation due to saltwater injection was more clearly detectable at frequencies from approximately 30 kHz to 100 kHz, whereas reconstructions obtained outside this range contained very limited useful information.

## 4. Discussion

The results confirm that Electrical Impedance Spectroscopy (EIS) and Electrical Impedance Tomography (EIT) are promising non-destructive techniques for fruit quality monitoring. The impedance variations observed during the 15-day storage period are consistent with physiological changes previously reported in the literature, such as membrane degradation, changes in water distribution, and variations in extracellular conductivity during ripening and senescence.

A key outcome of this study is the comparison between two-electrode and four-electrode configurations. The experimental results show that electrode-sample interface impedance and other measurement-system contributions can significantly affect low-frequency measurements in two-electrode setups, leading to an overestimation of the impedance values.

In contrast, the four-electrode configuration minimizes interface effects and provides more reliable measurements in the β-dispersion frequency range, which is particularly relevant for biological tissue characterization.

The equivalent circuit analysis also showed that fractional-order models effectively describe the electrical behavior of fruit tissues. Although the double-Cole model achieved the lowest interpolation error, the Hayden-based models provided a simpler physical interpretation of extracellular and membrane-related processes while maintaining satisfactory accuracy. The increase in extracellular resistance and the decrease in membrane capacitance may be consistent with the findings reported in [64]: during post-harvest storage, the tissue progressively loses cellular integrity and undergoes changes in its water/ion composition, resulting in a decrease in membrane capacitance and an apparent increase in extracellular resistance.

Preliminary EIT reconstructions further demonstrate the feasibility of non-invasive tomographic imaging using a limited number of electrodes and commercially available instrumentation. The limited amount of literature currently available for fruit applications indicates that further validation studies are required before EIT can be considered a mature tool for routine fruit quality assessment.

A practical limitation of the proposed EIS/EIT approach is the need for reliable electrode placement on fruit surfaces, particularly when multiple electrodes are required. Future developments should focus on flexible and wearable electrode technologies, automated positioning systems, and robotic integration to improve measurement repeatability and facilitate deployment in precision agriculture and postharvest monitoring applications.

Future work will focus on improving spatial resolution, optimizing reconstruction algorithms, extending the analysis to different fruit types, and integrating impedance techniques with portable and robotic sensing platforms for precision agriculture and smart food processing applications. In addition, multimodal approaches combining impedance measurements with complementary sensing techniques, such as optical, spectroscopic, hyperspectral, or tactile methods, represent a promising direction for improving the reliability and robustness of fruit quality assessment systems.

## 5. Conclusions

This work reviewed recent advances in Electrical Impedance Spectroscopy (EIS) and Electrical Impedance Tomography (EIT) for non-destructive fruit quality monitoring and presented experimental investigations on multi-electrode impedance measurements performed on yellow honeydew melon samples.

Although EIS and EIT address different measurement objectives, they share common physical principles, instrumentation, and electrode technologies. EIS provides frequency-dependent characterization of fruit tissues, whereas EIT extends impedance measurements to the reconstruction of spatial conductivity distributions, making the two techniques complementary approaches within the broader field of impedance-based sensing.

The results confirmed that impedance-based techniques are sensitive to physiological changes occurring during storage and ripening, particularly those related to membrane integrity and extracellular conductivity.

The comparison between two-electrode and four-electrode configurations highlighted the strong influence of electrode polarization on low-frequency measurements and demonstrated the improved reliability of four-electrode approaches for biological tissue characterization. Fractional-order equivalent circuit models, especially the double-Cole and Hayden-based models, provided accurate interpolation of the experimental spectra and enabled the extraction of meaningful parameters associated with tissue evolution over time.

Preliminary EIT measurements and reconstructions also demonstrated the feasibility of low-cost, non-invasive tomographic imaging using commercially available instrumentation and a limited number of electrodes. Although further optimization is required, the proposed approach shows potential for integration into portable sensing systems, robotic platforms, and precision agriculture applications.

Overall, EIS and EIT represent promising tools for real-time food quality assessment, offering advantages in terms of low invasiveness, portability, and compatibility with smart monitoring systems.

## Figures and Tables

**Table 2:** Absolute and percentage values of RMSE (and standard deviation) for considered equivalent circuit models.

Model Name	RMSE_1_/Ω	RMSE_2_/%	RMSE_3_/%
Single-Cole	2.39 (0.21)	1.77 (0.15)	4.60 (0.53)
Double-Cole	0.59 (0.12)	0.39 (0.05)	1.62 (0.33)
Fractional Hayden	2.40 (0.21)	1.80 (0.12)	4.62 (0.51)
Simplified Fractional Hayden	3.50 (1.02)	2.75 (0.92)	6.02 (1.51)

## Data Availability

The raw data supporting the conclusions of this article will be made available by the authors on request.
